# Barriers of Appropriate Antibiotic Prescription at PHCC in Qatar: Perspective of Physicians and Pharmacists

**DOI:** 10.3390/antibiotics10030317

**Published:** 2021-03-19

**Authors:** Nahla Sharaf, Ghadir Fakhri Al-Jayyousi, Eman Radwan, Shimous Mohamed Elamin Shams Eldin, Dhouha Hamdani, Huda Al-Katheeri, Khalid Elawad, Anjum Habib Sair

**Affiliations:** 1 Department of Strategic Planning and Performance, Ministry of Public Health, Doha P.O. Box 42, Qatar; nsharaf@MOPH.GOV.QA (N.S.); eradwan@MOPH.GOV.QA (E.R.); seldin@MOPH.GOV.QA (S.M.E.S.E.); dhamdani@MOPH.GOV.QA (D.H.); halkatheeri@MOPH.GOV.QA (H.A.-K.); 2 Department of Public Health, College of Health Sciences, QU Health, Qatar University, Doha P.O. Box 2713, Qatar; 3 Preventative Health—Health Protection, Primary Healthcare Corporation, Doha P.O. Box 26555, Qatar; kelawad@phcc.gov.qa; 4 Operations—Clinical Operations, Primary Healthcare Doha P.O. Box 26555, Qatar; asair@phcc.gov.qa

**Keywords:** antibiotics, antimicrobial resistance, primary health care, antibiotic prescription, antibiotic use, Qatar

## Abstract

The Ministry of Public Health in Qatar developed the NAP (National Action Plan to combat Antimicrobial Resistance (AMR) in collaboration with WHO Regional Office for the Eastern Mediterranean (WHO/EMRO). Among the major factors shaping AMR is antimicrobial prescribing and use. Tailoring Antimicrobial Resistance Program is a behavior change methodology that is utilized to adapt behavior change in relation to antimicrobial use. This study explores barriers of appropriate antibiotic (AB) prescription from the physicians’ and pharmacists’ perspectives at primary healthcare centers in Qatar. Data were collected from 50 participants across two PHCCs: 30 physicians and 20 pharmacists. Two different interview guides were constructed: One for physicians and one for pharmacists. In-depth, face-to-face, five focus groups were conducted and transcribed verbatim. Inductive qualitative analysis, involving discovering the themes in the interviews, was followed. Data were analyzed using constant comparative techniques. The Major themes arose from the analysis revealed that patients, practitioners mainly physicians, and the organization itself, played a role in shaping these barriers in the two primary healthcare centers. The findings would help develop and pilot behavior change interventions among patients, physicians and pharmacists with the aim of optimizing appropriate antibiotic prescription and use, which would support the implementation of the antibiotic stewardship program. Effective behavior change interventions should consider multiple factors including individual and organizational factors to optimize appropriate antibiotic prescription.

## 1. Introduction

In recent years antibiotic resistance (AR) has become a growing problem and one of the main contributors to antimicrobial resistance (AMR) which continues to pose a great threat to public health worldwide [1,2]. The development of AMR is a natural phenomenon. However, due to overuse and misuse of antibiotics in different sectors; human and animal health, aquaculture and agriculture. The rate at which AMR is occurring is exponentially increasing. This misuse threatens modern medicine and jeopardizes the safety of the routine treatment of infections.

Infections caused by resistant bacteria, viruses, fungi and parasites, especially multi-drug resistant organisms, can lead to severe illness, prolonged hospitalization, treatment failure, and higher healthcare cost, as well as an increased mortality rate [1,2,3]. Antimicrobial stewardship is key to optimizing the use of antimicrobial drugs and combat antimicrobial resistance. Stakeholders in the healthcare industry, healthcare organizations, physicians, pharmacists, and patients are essential elements to guard antibiotics and ensure effective use of the medicines in general [4].

The inappropriate use of antibiotics may arise from the interaction of many factors, namely prescribers’ knowledge, behavior and experiences, diagnostic tools, perceptions of physicians, pharmacists and patients in relation to the patient-prescriber and pharmacists interaction, and insufficient information and education provision from healthcare staff to patients. Moreover, there are several additional factors which further contribute towards this phenomenon including patient knowledge, beliefs and attitudes towards antibiotic use, self-medication, patients’ expectations, and patients’ experience with antibiotics are other contributing factors to the problem [5,6].

In terms of research studies conducted in the Gulf Cooperation Council (GCC) countries, which included Qatar, there were two significant reports which brought to light the emergence of antimicrobial resistance to the region [7,8]. Both studies demonstrated the high probability that antimicrobial resistance is predominantly consequence of the inappropriate prescription and overuse of antibiotics as well as pertinent issues concerning self-medication. Other factors included the lack of clear guidelines for the use of antimicrobials and lack of policies for restricting and auditing antimicrobial prescriptions in many GCC countries [7,8].

A study was conducted in 2009 at 50 primary healthcare centers in Kuwait to explore the practices and behavior of healthcare practitioners in primary care in relation to antibiotic prescription. The results of the study showed that almost four in ten prescriptions included an antibiotic as part of the required course of treatment for the patient [9]. In an-other study, Baadani et al. sampled 212 doctors in Riyadh, KSA and sought to understand their attitudes, knowledge and perceptions towards the prescription of antimicrobials. The study concluded that there is an alarming deficiency in the training of physicians concerning the prescription of antimicrobial drugs [10]. Further in-depth studies exploring such practices and behavior in relation to antimicrobial use in the region are required.

Nearly 30% of the antibiotics prescribed in the United States are unnecessary, according to the Centers for Disease Control and Prevention (CDC) [11]; 90% of total antimicrobial use are at outpatient settings, with more than half of these prescriptions being either unnecessary or inappropriate [12]. Another study in the UK showed that between 20 and 50% of all antimicrobial use was inappropriate [13]. In 2012, the European Centre for Disease Prevention and Control (ECDC) collated data from several countries from the European Union on both ambulatory and intra-hospital antibiotic use and found that 85–95% of the total antimicrobial use could be attributed to ambulatory antimicrobial consumption [13].

In Qatar, the use of antibiotics occurs mainly at the primary care level where approximately 300,000 antibiotic prescriptions are provided to patients annually [14]. ECDC compared non-hospital with hospital usage and found that the first is significantly more than the latter. At primary healthcare, the use of antibiotics exceeds 10 defined daily doses (DDDs) per 1000 population day, while the hospital use was three DDDs [15]. Therefore, in order to reduce such poor prescribing patterns of antimicrobials in primary care settings, it is critical to enable the adoption and implementation of established optimum prescription practices. However, a key current challenge is the number of variables which impact prescribers and dispensers of antibiotics, not only that but these variables differ according to factors such as geographical region, social circumstances, and the prevailing healthcare system which all play a role.

In recent years in Qatar, there has been a broad recognition of the issues around deficiencies in knowledge and general awareness of inappropriate practices of antibiotic use from healthcare professionals and the general public, however certain barriers continue to exist thereby prohibiting the current drive to promote optimum practice. Further to this awareness and in an attempt to bring about change in the indiscriminate use of antibiotics and associated development of bacterial resistance, a number of interventions has been recommended, which target the general population, health care providers, and regulatory authorities [3]. Furthermore, antibiotic usage and auditing practices may vary from year to year and from country to country which may not necessarily be explained by the incidence of the infections [15].

There are several key paths to the reduction of bacterial resistance. The first is through the adoption and implementation of prudent prescription and dispensing practices by both clinicians and pharmacists which means improving the diagnosis of bacterial infection and subsequently selecting the suitable antibiotic, route and dose. The second is the establishment of antimicrobial policies in order to support the health professionals responsible for making the decisions to prescribe the aforementioned drugs. This can be done through antimicrobial stewardship programs, which are interventions to encourage behaviors based on a more evidence-based approach. Such programs usually target the reduction in volume of prescribed antibiotics and are made up of different elements, such as audit and feedback, clinician and patient education, and use of point-of-care tests [16]. Pharmacists are pivotal in ensuring effective and safe administration of these medications through their role with prescribers and patients.

Careful measurement of the mediators of behavioural change alongside the theory allows for the demonstration of how an intervention works and ensures that any unintended consequences of these often complex interventions can be understood and explained [16]. In Qatar, the Ministry of Public health has developed national action plan to combat antimicrobial resistance in collaboration with WHO. Tailoring Antimicrobial Resistance Programs (TAP) is a behavior change methodology utilized in this study aiming at changing behaviors in relation to antibiotic use. The TAP intervention is step-by-step methodology that includes situation analysis, behavior analysis, and the development of a strategic behavior change intervention with an evaluation of the intervention.

To our knowledge, there are no studies in Qatar explaining AMR behavior drivers among physicians and pharmacists. Thus, this qualitative study was designed to explore barriers and motivators of appropriate antibiotic prescription from the physicians’ and pharmacists’ perspectives at primary health care in Qatar. The results of this study would establish an effective startpoint for healthcare providers in terms of knowledge, attitude and perception towards the use of antimicrobial drug prescription. Accordingly, such a baseline will support stakeholder assessment and review of the suitability of the currently deployed approaches to antibiotic stewardship. The baseline would also provide a strong basis to facilitate in the implementation of complex multidimensional interventions across the healthcare sector.

## 2. Results

The majority of the participants in our study perceived antimicrobial resistance (AMR) as a serious, global public health problem. They varied in how they perceived AMR in Qatar; few of them agreed that it is a huge problem in the country; however, most of the participants explained that it is under control. They explained that the most common health problems or infections that patients presented with in the centers included upper respiratory tract infections (URTI), tonsillitis, pharyngitis, otitis media, Urinary Tract Infection (UTI) and gastroenteritis. Participants also mentioned that the most common antibiotics they prescribed were beta lactams in general, including Amoxicillin-/clavulanate potassium, Amoxicillin, and Cefuroxime, Macrolides as Clarithromycin, and Metronidazole.

Participants showed that a major factor shaping AMR is the inappropriate prescription and use of antibiotics and they explained the barriers that prevented the appropriate prescription and use: “I think it is a huge problem. Historically, Antibiotics used to be given to children as smarties. Everybody is prescribing amoxicillin-potassium clavulanate and we are at the stage where it is not working anymore”. The Major themes arose from the analysis revealed that patients, practitioners mainly physicians, and the organization itself, played a role in shaping these barriers in the mentioned two primary healthcare centers. (See Table 1).

### 2.1. Patient’s Role in Preventing Appropriate Antibiotic (AB) Prescription and Use

#### 2.1.1. Patient’s Pressure to Prescribe AB

All physicians agreed that patients would pressure them to prescribe AB even when there is no need for it: “Some patients come with a fixed idea; they want AB, and they will insist. They think the doctor is here only to give AB, so you sometimes feel you are working at a supermarket”. They explained that some patients came to them with common cold symptoms, cough, and sore throat and they insisted that they need AB to alleviate the symptoms. Physicians would educate patients that it is a viral infection, need time to recover and try to explain to them the unnecessity of prescribing AB. Unfortunately, some patients when they do not have an antibiotic, they would complain to the administration in the center and make an appointment with another physician who might prescribe it. Physicians claimed how this might influence their reputation.

“Patients with cough come to the center just for antibiotics. Patient come with sore throat and if there is no antibiotic, there is a lot of work to be done. You need to explain it is viral and you don’t need antibiotics so lot of work… I think the main issue is the patient perception of the whole issue because I have seen many patients who come here and we explain that it is a viral infection which may take 5–7 days sometimes 10 days and try and explain to them whether they understand or not.”

The majority of the pharmacists (70%) agreed that patient’s pressure can be considered one of the barriers of appropriate AB prescription. They explained that if the physician did not prescribe the AB, they might come to them, as one participant mentioned: “Less educated people, they come directly to the pharmacy and ask to dispense certain type of AB (yellow capsule) that they used before. If we refused, they may make trouble…He starts blaming the doctor and argue for his AB”. Pharmacists mentioned that patients would also argue the type of the AB; if a physician prescribed Amoxicillin, they might consider it a weak AB and ask for Augmentin. They also argued about the dose: “Sometimes they argue about the concentration of AB, about the dose. If the doctor gives him 250 mg, he would ask for 500 mg. Or the patient gets angry and starts to ask the doctor for Cefixime 400, or why you don’t give 500 mg?”

Participants talked about the demographics of patients who might pressure prescribing unnecessary AB in the centers. They mentioned that patients with low education across all nationalities, elders and parents, who bring their children once or twice a month, were very demanding to prescribe AB.

#### 2.1.2. Patient’s Behavior in Regards to AB Use

Physicians (60%) and pharmacists (60%) mentioned that patients might follow inappropriate AB use and AB self-medication. Patients might start the AB before consulting the physician: “Sometimes you come across a patient who has already taken antibiotics before coming to see you. They have already had it from abroad or another hospital in the country, they started before they came to see you”. Other patients will not complete the AB course; they would stop when they feel better and might keep it for next sickness, and/or share it with another family member or a friend. In addition, patients might use these AB kept at home when they have Common cold symptoms and even only headache

“They only take half the course and keep the rest for next time when they feel sick. This leads to another problem; that is the duration of the antibiotic. Some people take it only for two days, then they don’t complete the whole course and they keep the rest to use later.”

### 2.2. Practitioners’ Role in Preventing Appropriate AB Prescription and Use

#### 2.2.1. Physician’s Malpractice in Regard to Prescribing AB

Majority of Physicians (75%) explained how some of their colleagues lack the needed knowledge and appropriate practices about narrow- spectrum AB and when they need to prescribe them: “Everybody is prescribing Augmentin and we are at the stage where it is not working anymore… Education around giving narrow-spectrum antibiotics is needed. Here, Augmentin you can get it any time”. Aligning with this, pharmacists (70) also mentioned that physicians might prescribe unnecessary AB, broad and strong ones.

#### 2.2.2. Hard to Clinically Differentiate between Viral and Bacterial Infections; Yet, Not Requesting Microbiological Investigation

Half of the physicians (50%) mentioned that they are challenged to decide about the type of infection and thus decide if a patient needs AB or not. One physician explained: “There is nothing in medicine that is black and white. There are many gray areas. Sometimes you have patients walk in and I’m not 100% sure whether this is a viral or bacterial. You prescribe AB to be on the safe side”. Another physician explained that when a patient stopped by with a fever for 2 or 3 days, this is a typical viral infection, but it is very difficult to send him back home without an AB, especially if he/she is a child who might develop secondary bacterial infection and would be a risk to not to prescribe AB. Others mentioned that there are atypical cases, which made them even more challenged to decide:

“But there are some atypical cases that we need to cautious when diagnosing it as viral URTI and then patient comes twice not resolved and the chest X-Ray shows bilateral pulmonary infiltrates, so we need to be aware of atypical bacterial infections. There are some criteria for bacterial infections and old people with comorbidities, renal impairment, and immunosuppression.”

Physicians explained that they can run some rapid diagnostic tests to find if the infection is bacterial or not and justify the need to prescribe AB; however, because of the workload at the center, they would not perform the test: “The only problem is that doctors don’t have so much time to follow it. If we have a focal person like a nurse who is trained to take the sample, throat swab, do lab test and Point of Care POC testing, we will do it. Essays are available for viral infections, but nobody actually request it”.

Pharmacists (50%) also mentioned that physicians would not request culture to test AB sensitivity. They explained that a patient might come back to the center, still sick and not responding to the AB he took, because he was resistant to multiple AB. However, they did not know to what AB the patient was resistant as the physician did not ask to perform the sensitivity/susceptibility test.

#### 2.2.3. No Clear Understanding/Following of the Clinical Guidelines

Physicians (30%) mentioned that the clinical guidelines about AB prescribing were not easily accessed to them, there were large amount of information to comprehend, and they should be revised as one physician explained:

“For example in UTI, we only have nitrofurantoin, what if it doesn’t work? I need to have another choice, and then again we end up prescribing ciprofloxacin or amoxicillin-potassium clavulanate for UTI which can be treated with trimethoprim that is a narrow spectrum AB. So, I think we need to revise the availability of AB and the guidelines for AB prescribing. I had a look at the guidelines a couple of months ago; it’s confusing, it’s huge.”

Pharmacists (20%) also talked about how the implementation of some guidelines such as the process of delayed AB prescription were not clear to them, as one participant mentioned: “Some pediatric cases with mild infection, doctor gives the strong AB like Zinnat or Suprax and tell the mother if the temp did not come down, give him AB within 48 h. But, the mother sure will not wait and will give AB directly”.

A few physicians and pharmacists agreed that the national clinical guidelines were not popular at the centers, and how some physicians did not follow them. One pharmacist mentioned that a lot of AB dispense was unnecessary and some physicians create their own regimens, which were not related to any guidelines.

#### 2.2.4. Limited Physician-Pharmacist and Physician-Patient Communication Related to AB Prescription and Use

Pharmacists (70%) mentioned that the final decision in regard to AB prescription referred to the physician. They elaborated that they may discuss some cases with physicians such as AB sensitivity, the appropriate choice of AB, and the AB formula; however, some doctor might refuse to make any modifications:

“Yes, another issue if we ask the doctor to change AB to a more appropriate one, in this case he may refuse. Sometimes the formula is not accurate (wrong) one doctor wrote Augmentin and cephalosporin. We try to discuss and correct the formula, in this case he accepted and changed it.”

Pharmacists also mentioned that physicians do not communicate with patients about the right dose of the AB, the duration and the side effects. They explained that physicians do not educate patients about appropriate use of AB.

### 2.3. Organization’s Role in Preventing Appropriate AB Prescription and Use

#### 2.3.1. AB Prescribing Regulations

Physicians (75%) mentioned that currently antibiotics have to be prescribed in the government and private sectors; however, there is no restrictions on when to prescribe. They explained that in primary healthcare, the organization is encouraging individuals to seek healthcare with any viral symptoms, they are actually considered priority and should be provided with the best treatment and AB would be prescribed:

“We are encouraging patients who have any viral symptoms, they have this concept that they have to see the doctor. Because, we welcome patients; if you got cough and flu the most common presentation, you are the priority, you are the priority, you are the emergency, you need to be seen today.”

In addition, they mentioned how it is the same scenario in other government health organizations, which are considered secondary and tertiary healthcare settings and in emergency departments. They explained that: “Last week I saw a patient who went to the [government hospital] with sore throat and was given Augmentin, and asked me: Do I need AB? Sometimes, doctors in emergency have a huge workload so give the patients the AB to move on to next patient”.

Physicians and pharmacists (50%) mentioned when patients are unable to have AB from the primary healthcare centers and other government health organizations, they would seek the private healthcare to get AB prescription. Antibiotics are expensive, so patients who have health insurance will seek a private health center to get the prescription, as one pharmacist explained; “No way to go to private clinic and not take AB, they have insurance, they pay for that”. Thus, although they need prescription, which would put some control, still there are ways where they can get antibiotics”.

Finally, both also talked about a restriction policy in primary healthcare in prescribing specific broad spectrum AB, which would lead to inappropriate prescription. They explained how family physicians do not have the authority to prescribe specific AB according to the sensitivity test, because only consultants are allowed to request the test and prescribe these antibiotics.

#### 2.3.2. Data about AB Resistance Patterns and Availability of Narrow Spectrum Antibiotic

Participants from the physicians’ (30%) and pharmacists’ (20%) focus groups mentioned that the organization do not share any information about AB resistance patterns, nor any antibiogram data with them. They also explained that the narrow-spectrum AB were not available in the centers, thus they ended up prescribing whatever they have in their hands: “Absolutely, as we said before availability of narrow spectrum AB that would be one factor. Here, the patient comes and we don’t have much choice, so we write what is available”.

#### 2.3.3. Workload and Restricted Time of Consultation

The majority of the physicians (70%) talked about how they are overwhelmed with the workload and the number of patients they see every day, which prevented them from providing the needed education for patients in regard to AB use:

“We get exhausted, we have 20–30 patients, 5 general patients and 25 URTI, then bringing them in and talking to them, they exhaust us. A nurse goes down to those five patients, which are the actual patients we don’t get that much time and energy to educate them on their chronic conditions and AB.”

Physicians explained that because of the time constraint, they were also unable to request the rapid diagnostic test that would help them decide about the type of infection, then prescribe the effective AB:

“It takes about 5 min and the lab will take about 10–15 min, and the result will come after ½ an hour… So, better to have some mechanism that if you want to confirm your diagnosis. A focal staff nurse has been trained like that and she gets the results and inform doctor and if the patient can wait. That will decrease the burden of AB prescription by at least 15–20%.”

#### 2.3.4. Management Response to Patient’s Complaints and Patients’ Rights

Physicians (30%) and pharmacists (20%) mentioned that PHCC management would always support patients and be on their side. They explained that if a patient complained to the center administration that a physician should have prescribed an AB, but did not, then this might put the physician in trouble and at the end they would prescribe the AB for the patient. One pharmacist/physician mentioned:

“Some doctor not respond to patient and explain to patient it is viral infection, and no need for Ab, but the patient will complain to administration. System will say that the doctor is making problems with patient/trouble maker. So, power of the patient is stronger than us, the system is not supporting the doctors. So, usually we said why I put myself in trouble, and will write what the patient needs.”

Physicians also elaborated how the organization is giving the patients the right to switch to another physician if they were not satisfied from the treatment. So, when a patient was expecting an AB prescription, and the physician did not prescribe one, he/she would change the physician: “…they have the right to change and they have the right to choose the DR. They say I need to be seen by DR [doctor name]. By law here they have the right. Even if it is not the same team”.

#### 2.3.5. Pharmacists’ Authority Is Limited

The majority of the pharmacists (60%) complained that they have limited role in influencing the AB prescription process. They elaborated that they were not allowed to do auditing for AB, they even did not have any training about AB auditing. They explained how they should monitor the prescription process, but there was no time for monitoring, and they did not have the authority for monitoring, nor the right to generate any report from the system. Pharmacists can only make suggestions, but no authority for actions, which may be considered a barrier for appropriate AB prescription.

## 3. Discussion

In this qualitative study, focus groups were utilized to determine the common barriers faced by physicians when prescribing and pharmacists when dispensing appropriate antibiotics in the PHCC setting. Primary care is a critical player in addressing the problems of inappropriate antibiotic prescribing. The results revealed three main themes that negatively impact the prescribing and dispensing of antibiotics to patients which are: Physician’s knowledge and practice, clinical guidance time, patient pressure and organizations regulations.

As described by Borek (2020), time is one of the biggest barriers to the appropriate prescribing of antibiotics in a primary care setting [17]. Limited time with patients reduces the overall ability of a physician to communicate effectively [18]. The focus groups revealed that physicians felt that the consultation time coupled with the number of patients who needed to be seen per day led to the incorrect prescribing of antibiotics, as well as other medication. This is due to two factors, firstly, a short consultation time prevents physicians and pharmacists from raising awareness about antimicrobial resistance and providing education to their patients regarding the nature of when antibiotics are acceptable. Secondly, physicians were unable to request the rapid diagnostic test which would reduce the risk of diagnostic uncertainty. In doing so, physicians were faced with the pressure to diagnose and treat within a short time frame. In addition to not being able to perform the rapid diagnostic test, the pharmacists also stated that due to the time constraints, physicians were also unable to perform the sensitivity/susceptibility which is critical for tracking individuals and whether they are resistant to particular antibiotics. Both tests provide much needed data for the health centers.

Another well-documented barrier to preventing the appropriate antibiotic prescribing which is found in other studies has been the influence of patient pressure [19]. Jeffs et al. (2020) state that patients would often pressurize their physicians to prescribe antibiotics based on their preconceived notion of a previous experience of feeling better [20]. This patient pressure has been highlighted in the PHCC practices by physicians across Qatar. A patients’ pressure arises in two distinct forms, either they would demand antibiotics regardless of whether the physician stated it was not necessary, or the patient would demand a higher/stronger form of antibiotics. A patient’s expectations, which can only be addressed with time and education, has been shown to heavily influence prescribing habits [18].

According to the focus groups, in circumstances when the physician did not prescribe antibiotics, the patient would as a result either book an appointment with an alternative physician at the health center, complain to management, or demand antibiotics straight from the pharmacists. In doing so, the physicians expressed that their reputation would be impacted negatively if the patient caused trouble. This fear demonstrates an underlying theme that there is an overall pressure to ensure a positive patient experience for those who visit the health centers above all else. This is a common finding across other studies, e.g., Ashworth et al. who state that, ‘antibiotic prescribing was a significant determinant of patient experience [21]. Zetts et al. (2020) state that because of the pressure, physicians understandably feel that resistance can be futile, especially when the patient can seek antibiotics from alternative healthcare providers [22].

A final barrier which has been found across multiple studies is that those in a position of authority to prescribe do not feel that they have the support in the form of clinical guidelines and therefore there is a lack of standardization in antibiotic prescribing across all healthcare sectors [23]. The focus groups revealed that although clinical guidelines are available, not all physicians are aware of their contents, and this is due to the lack of appropriate implementation of the guidelines across all healthcare sectors, as well as the fact that the physicians do not feel they have the time to comprehend its contents in its entirety. It was reported that ultimately more training should be provided to those in a position to prescribe and specifically, how to implement recommended techniques to reduce inappropriate antibiotic prescribing, such as the delayed antibiotic prescription approach. The process of delayed antibiotic prescription is a well-known tactic utilized globally to reduce antibiotic misuse, to the extent that Sargent et al. (2017) reports antibiotic use reduces by up to 60% [24].

In addition to the lack of appropriate implementation of clinical guidelines, the focus groups also reported that there was a lack of availability of educational resources, audit reports and antibiogram data. If the prescriber is more aware of their prescribing habits, they would be more likely to improve their prescribing practice, especially if regulations were applied to all providers across Qatar [19].

The factors described above are those that are known and well documented in international studies. However, interwoven with these are additional influencing factors that require highlighting to better understand the antibiotic prescribing landscape in Qatar:

- Physician/Pharmacist background/culture—PHCC employs a myriad of professionals from significantly diverse backgrounds. This is especially true for Physicians. Hence, the different backgrounds contribute to different prescribing practices and decision making.
- Cost- The antibiotics in PHC are currently funded while they are expensive at community pharmacies, so the patients come to PHC center to request their antibiotics at low cost.
- Workload—with the ever-expanding role of Primary Care Family Physicians to provide integrated care services, more and more secondary care-based services are being delivered within the Primary Care setting to improve access to services for patients across the care continuum. This additional workload reduces the time staff can spend on explaining AMR to patients and to avoid antibiotic prescribing.
- Electronic Health Record (Cerner)—Qatar is in a unique position in that all health sectors—hospitals (secondary, tertiary), primary care centers as well as schools (except private health facilities) are all connected electronically providing access to the single patient health record. Although this has significant patient safety advantages, the challenge for staff is the time taken to complete electronic documentation versus patient directed time. This (amongst other factors), reduces the opportunities for physical examination of patients and the time required to explain AMR to patients requesting antibiotics
- Circumventing the system—where a Physician has the time, knowledge and patience to explain the rational for not prescribing a course of antibiotics, if the physician insists on not prescribing antibiotics, the patient will simply leave and re-book another appointment with another physician who will be pressured to prescribe. Such scenarios are common and cause demotivation for staff trying to enforce organizational restrictions on antibiotic prescribing.
- Patient/Public factors—Pressurizing Physicians to prescribe antibiotics to order—patients have significant influence over staff employed from foreign countries who value their employment and cannot afford to have any complaints made against them for refusing a patient request and Lack of patient awareness of AMR and the need for rational antibiotic use.

In order to address one of the main barriers which is that there is no widespread enforcement of the clinical guidelines, the guidelines should be revised to ensure they are up to date and relevant, and then implemented alongside training for all healthcare prescribers. By creating the nationwide foundation provider’s competency and knowledge, the individuals will be able to better implement methods, such as the delayed prescription process, to their patients [24]. In addition, staff will also be able to manage patient expectations in the process [25]. The clinical guidelines will ensure a unified front from all healthcare providers in Qatar, delivering a standardized service in both primary care and smaller practices. This training and enforcement of the guidelines will help to empower providers.

In conjunction with the clinical guidelines, prescribers should have easy access to information and regular audit reports which include antibiogram data. Mercer et al. (2019) state that audit reports are a valuable source of information that “reinforce not to overuse antibiotics”. Providing direct feedback to the prescriber has been demonstrated to successfully reduce antibiotic prescribing [26]. By providing this data, in addition to providing training to pharmacists specifically on how to conduct audits, information would consequently become a facilitator to appropriate antibiotic prescription. This would ensure that all those in a position with authority would fall under the same umbrella of responsibility [22].

To successfully reduce patient pressure, Zetts et al. (2020) state that a patient’s awareness and understanding of antibiotic prescription, needs to increase [22]. By educating a patient successfully, the patient will be less likely to pressurize their physician, in addition, the patient would also have the added understanding of how to correctly take antibiotics. Introducing education programs for providers as well as patients, will help to address the gap in knowledge [25]. Education on antibiotic prescribing is a core element of the CDC Framework and it can be provided on electronic learning platforms to help increase uptake [27].

To reduce diagnostic uncertainty, physicians and pharmacists suggested the training of nurses which would allow for tests to be conducted in a time beneficial manner. By extending this authority, physicians would be able to test more patients and thus reduce the levels of diagnostic uncertainty within the primary care setting.

For these recommended solutions to be successful, a multipronged approach is needed whereby there is an increase in the collective responsibility across all healthcare providers and healthcare workers that is enforced with government support and key stakeholders [19]. Based on multiple studies, including Qatar’s, healthcare providers often feel that no one single individual can address the incorrect prescription of antibiotics, but rather it is a responsibility shared by all [20]. An increased collective effort supported by national guidelines in conjunction with education will help to reduce the rise of antibiotic misuse and incorrect antibiotic prescription [26].

Considering the findings, a multipronged approach will be adopted to formulate an intervention aiming at improving the awareness and changing behavior of the public and healthcare providers regarding the use and prescription of antibiotics. The intervention will include widespread enforcement of clinical guidelines, promote the use of rapid diagnostic testing, provide antibiogram specifically for primary care, improve clinical audit on antibiotic use and feedback to physicians and pharmacists and enhance the role of pharmacists in stewardship programs.

A limitation of this study has been the use of focus groups. Focus groups rely heavily on assisted discussion to provide results from the mediator, and as a result, discussions can be influenced impacting the results [28]. To help alleviate these limitations, the mediator was not affiliated with the participants. This also removed the bias from the focus group. However, in our study, we reached data saturation. All the focus group discussions (FGDs) were conducted in English language which may be the second language for many participants. However, all participants are highly educated in English language and did not find any issues in understanding the questions and participating in the discussion.

## 4. Materials and Methods

### 4.1. Data Collection

Two centers were selected for participants’ recruitment. They were chosen by PHCC operational management, because they were not involved in previous research investigating antibiotic prescribing, nor public practices on antibiotic usage compared to other primary health care centers.

After receiving approval from the Institutional Review Board (IRB) at Primary Healthcare Corporation to conduct this research study, a criterion sampling strategy was used. The criteria included: (1) Being a physician (family Medicine physician, general practitioner specialist or consultant) or a pharmacist from the two selected primary health care centers, and (2) Being involved/or allowed to prescribe and/or dispense antibiotics. The managers of the two centers called for participation through e-mail lists of the target audience. Fifty participants were recruited (30 physicians and 20 pharmacists) from the two centers.

Next, participants signed consent forms, and in-depth, face-to-face focus groups were conducted in the two chosen centers. Two different interview guides were constructed: one for physicians/prescribers and another one for pharmacists/dispersers. The questions explored the physicians’ and pharmacists’ perception regarding the factors (barriers and motivators) influencing the prescription of antibiotics at PHCC. In addition, the participants were asked about their opinion regarding how to reduce the prevalence of antimicrobial resistance in Qatar. Each focus group lasted approximately 45 min–1 h and was digitally audio-recorded. The interviews were transcribed verbatim by using Scribe.com software.

### 4.2. Qualitative Analysis

Our analyses utilizes a constant comparative method [29]. Members of our research team took part in the analyses process. During the initial phase of the open coding process, the focus group interviewers and observers identified themes based on focus group transcripts and their notes. Inductive qualitative analyses was used to discover themes in the responses, and predetermined themes were sought. Pieces of data were compared for similarities and differences. Each transcript was coded and new themes were added to the codebook as they emerge. Constant comparisons were conducted to differentiate one theme from another and to identify dimensions of each theme. As date is added, themes were added and modified as needed. Finally, the themes were combined into a coherent textural description of the phenomenon.

A different research team member who was not been involved in the focus group process reviewed the transcribed data. Once data were reviewed individually, the team met to discuss commonalities in the themes identified. In the second phase of axial coding, we examined how the themes compared across the different focus groups.

## 5. Conclusions

Primary health care physicians and pharmacists recognize multiple barriers for the appropriate use of antibiotics that include patients’ pressure, patient behavior in using antibiotics, physicians’ knowledge and practices and lack to appropriate implementation of guidelines, lack of specific antibiogram for primary care, workload and restricted time of consultation. Interventions targeting healthcare system, physicians, pharmacists and the general population are needed to overcome the barriers and change behaviors around using antibiotics.

## Figures and Tables

**Table 1:** Narrative Theme Dispersion.

Theme	Physicians (%)	Pharmacists (%)
Patient’s role in preventing appropriate antibiotic prescription and use		
Patient’s pressure to prescribe AB	100	70
Patient’s behavior in regards to AB use	60	60
Practitioners’ role in preventing appropriate AB prescription and use		
Physician’s malpractice in regard to prescribing AB	75	70
Hard to clinically differentiate between viral and bacterial infections; yet, not requesting microbiological investigation	50	50
No clear understanding/following of the clinical guidelines	30	20
Limited physician-pharmacist and physician-patient communication related to AB prescription and use	-	70
Organization’s role in preventing appropriate AB prescription and use		
AB prescribing regulations	75	50
Data about AB resistance patterns and availability of narrow spectrum antibiotic	30	20
Workload and restricted time of consultation	70	40
Management response to patient’s complaints and patients’ rights	30	20
Pharmacists’ authority is limited	-	60
